# Development of a predictive model for metachronous liver metastasis in gastric cancer

**DOI:** 10.3389/fonc.2025.1603471

**Published:** 2025-08-18

**Authors:** Siyuan Wang, Gaozan Zheng, Fengsu Wu, Ye Tian, Xinyu Qiao, Xinyu Dou, Hanjun Dan, Guangming Ren, Liaoran Niu, Pengfei Wang, Lili Duan, Yumao Yang, Jianyong Zheng, Fan Feng

**Affiliations:** ^1^ Department of Digestive Surgery, Xijing Hospital of Digestive Diseases, Fourth Military Medical University, Xi'an, Shaanxi, China; ^2^ Institute of Anal-Colorectal Surgery, The 989th Hospital of the Joint Logistics Support Force of People's Liberation Army (PLA), Luoyang, Henan, China; ^3^ Department of Gastroenterology, Pingdingshan Medical District, 989 Hospital of People's Liberation Army (PLA) Joint Logistics Support Force, Pingdingshan, Henan, China

**Keywords:** gastric cancer, monocyte, lymphocyte, metachronous liver metastasis, nomogram

## Abstract

**Background:**

Patients with metachronous liver metastasis (MLM) in gastric cancer generally have a poor prognosis. Early detection and accurate prediction of MLM are crucial for improving clinical outcomes. This study aims to identify the risk factors for MLM through clinical pathological parameters and develop a predictive model for MLM in gastric cancer.

**Methods:**

A retrospective analysis of 1248 gastric cancer patients who underwent radical surgery between December 2016 and December 2020 was conducted. Patients were randomly divided into training (70%, n=873) and validation (30%, n=375) datasets. The optimal cutoff values for the continuous variables were determined using the Youden index. Univariate and multivariate logistic regression analyses were used to identify risk factors for MLM. A nomogram was developed based on the results of multivariate analysis. The model’s value was validated through receiver operating characteristic (ROC) curves, calibration curves, and decision curve analysis (DCA).

**Results:**

The incidence of MLM was comparable between the training (10.3%, 90/873) and validation set (9.9%, 37/375). The optimal cutoff value was 3.315ng/ml for preoperative alpha-fetoprotein (AFP) level, 16.275U/ml for preoperative cancer antigen 125 (CA125) level, 0.280×10^9^/L for monocyte count and 1.430×10^9^/L for lymphocyte count, respectively. Univariate analysis showed that age, tumor size, pathological type, surgical method, T stage, N stage, TNM stage, neural invasion, lymphatic vascular invasion, number of lymph nodes harvested (LNH), preoperative total protein (TP), hemoglobin (HB), albumin (ALB), preoperative carcinoembryonic antigen (CEA), preoperative cancer antigen 19-9 (CA19-9), CA125, AFP levels, monocyte count, lymphocyte count, red blood cell (RBC) count and platelet count were considered as potential variables. Multivariate logistic regression analysis indicated that T stage, N stage, monocyte count, lymphocyte count, preoperative AFP and CA125 levels were independent predictive factors for MLM. The identified risk factors were further used to develop a predictive nomogram for MLM. The nomogram exhibited robust discriminatory performance, with an area under the curve (AUC) of 0.859 in the training set and 0.803 in the validation set. Moreover, the nomogram demonstrated excellent calibration and significant clinical utility.

**Conclusion:**

This study successfully developed a predictive nomogram for MLM in gastric cancer. Besides conventional parameters, we identified and incorporated peripheral blood monocyte and lymphocyte counts as novel predictors, demonstrating their independent predictive value. Integrating these factors into nomogram could enhance predictive accuracy of MLM.

## Introduction

Gastric cancer ranks as the fifth most prevalent malignancy and third leading cause of global cancer mortality (1). Despite the high incidence of gastric cancer, most patients are diagnosed at advanced stages with unfavorable prognoses due to the lack of distinguishing clinical indications (2). The liver is one of the most frequent sites of distant metastasis in gastric cancer (3–5). Critically, patients developing liver metastasis exhibit extremely poor prognosis, demonstrating a 5-year overall survival rate below 10% (6). Liver metastasis can be classified into two categories based on the timing of its occurrence following surgery: synchronous liver metastasis (SLM) and metachronous liver metastasis (MLM). According to international consensus, MLM refers to the occurrence of liver metastasis more than six months surgery (7). Approximately 2.0% to 9.9% of gastric cancer patients develop SLM, and up to 37% of gastric cancer patients develop MLM (4). Early detection and accurate prediction of MLM are crucial to improving outcomes in gastric cancer. Therefore, exploring the predictive factors of MLM in gastric cancer is very important.

Although extensive researches have been conducted on SLM in gastric cancer (8–10), studies focusing on MLM remain limited. Several clinical factors, including age, gender, T stage, N stage, Lauren classification, tumor size, histological type, and surgical approach, have been identified as potential factors for SLM (11). However, research on MLM, particularly its underlying mechanisms and risk factors, remains in its early stages.

Peripheral blood cells, including neutrophils, lymphocytes, monocytes, etc. could reflect the inflammatory status and immune response capacity *in vivo (* 12), holding significant value in the prognostic assessment of gastric cancer (13). Previous researches have predominantly focused on the analysis of ratio indices, such as neutrophil-to-lymphocyte ratio and platelet-to-lymphocyte ratio (14, 15). However, these composite metrics may obscure independent prognostic contributions of individual cell subsets. Critically, analyses based on absolute blood cell counts for predicting MLM in gastric cancer remain lacking.

Based on this, we aimed to develop a predictive model for MLM in gastric cancer, incorporating clinical pathological features, preoperative tumor markers, and peripheral blood cell counts. This model could provide clinicians with a reliable tool for early identification of patients at high risk for MLM, ultimately enabling more personalized treatment strategies and improving patient outcomes.

## Materials and methods

### Patients

This retrospective study enrolled patients who received curative gastrectomy in the Department of Digestive Surgery, Xijing Hospital of Digestive Diseases from December 2016 to December 2020.

The inclusion criteria were as follows (1): histologically verified gastric adenocarcinoma (2); no history of prior malignancy; (3) underwent R0 resection; (4) availability of complete clinical data; and (5) a minimum of 36 months of surveillance for non-MLM patients. The exclusion criteria were as follows: (1) liver metastasis occurring within 6 months after surgery; (2) synchronous distant metastasis at diagnosis; (3) subsequent non-liver metastatic progression; (4) incomplete follow-up records. Finally, the cohort (N=1248) comprised two groups: (1) the MLM group, defined as patients with liver metastasis occurring more than six months post-surgery (N=127), and (2) the non-MLM group, defined as patients without distant metastasis within 36 months post-surgery (N=1121). The study was conducted in accordance with the Declaration of Helsinki and received ethical approval (KY20232248-C-1) from the Ethics Committee of the Xijing Hospital of the Fourth Military Medical University.

### Clinical information

Clinical data, including gender, age, tumor location, tumor size, pathological type, surgical method, T stage, N stage, TNM stage, neural invasion, lymphatic vascular invasion, LNH, TP, HB, ALB, CEA, AFP, CA125, CA19–9 levels, as well as neutrophil, monocyte, lymphocyte, eosinophil, basophil, RBC, platelet counts from preoperative peripheral blood were collected through a retrospective review of medical records. Perioperative blood samples were obtained within 7 days before surgery.

### Clinical follow-up

A standardized clinical surveillance was performed every 3 months throughout the initial 3 years, followed by 6 months surveillance thereafter. Liver metastasis was diagnosed through contrast-enhanced CT. Non-MLM patients were monitored for a minimum of 3 years post-surgery. The follow-up period commenced from the day after surgery until the detection of MLM or the endpoint of the follow-up.

### Statistical analysis

Statistical analysis was conducted using R version 4.4.1 and SPSS version 27.0. To simplify the analysis and facilitated interpretation of results, continuous variables were converted to categorical variables. The optimal cutoff values for age, tumor size, LNH, TP, HB, ALB, CA125, CA19-9, CEA, AFP levels, neutrophil, monocyte, lymphocyte, eosinophil, basophil, RBC, platelet counts were determined by maximizing Youden index through ROC curve analysis (16).​​ The thresholds were identified corresponding to the maximum Youden index as the definitive cutoff value. Categorical variables were expressed as frequencies and percentages, with chi-square tests used. Univariate analysis was initially used to evaluate risk factors for MLM. Variables with a p-value < 0.1 in univariate analysis were then applied to forward stepwise multivariable logistic regression to identify independent predictors for MLM. A nomogram was developed based on the multivariate regression model. Its predictive accuracy was assessed using discrimination (AUC) and calibration through internal validation. Bootstrap resampling (1000 resamples) was applied separately to training and validation sets. Calibration plots were used to compare observed and predicted probabilities, while clinical utility was assessed through DCA curves. Statistical significance was set at p<0.05 for two-tailed tests.

## Results

### Baseline clinicopathological variables of the study population

The clinicopathological characteristics were summarized in **Table 1**. A total of 967 males and 281 females were included in the cohort, with a median age of 59 years. The median follow-up duration was 67 months (range 7-88). The cohort was randomly assigned to a training set (n=873) and a validation set (n=375) at a ratio of 7:3. The incidence of MLM was comparable between the training (10.3%, 90/873) and validation set (9.9%, 37/375). The optimal cutoff values for age, tumor size, LNH, TP, HB, ALB, CEA, AFP, CA125, CA19-9, neutrophil, monocyte, lymphocyte, eosinophil, basophil RBC, and platelet counts could were 57 years, 3.900 cm, 30, 67.100g/L, 104g/L, 40.700 g/L, 6.450ng/ml, 3.315ng/ml, 16.275U/ml, 24.735 U/ml, 8.960×10^9^/L, 0.280×10^9^/L, 1.430×10^9^/L, 0.060×10^9^/L, 0.030×10^9^/L, 4.480×10^12^/L and 281×10^9^/L, respectively.

**Table 1:** Characteristics of the patients in the training cohort and validation cohort.

Characteristics	Overall (n=1248)(%)	Training (n=873)(%)	Validation (n=375)(%)	X^2^	P
Gender				2.439	0.118
Male	967 (77.5)	687 (78.7)	280 (74.7)		
Female	281 (22.5)	186 (21.3)	95 (25.3)		
Age (year)				1672	0.196
<57	528 (42.3)	359 (41.1)	169 (45.1)		
≥57	720 (57.4)	514 (58.9)	206 (54.9)		
Tumor location				3.357	0.340
Upper third	365 (29.2)	265 (30.4)	100 (26.7)		
Middle third	301 (24.0)	215 (24.6)	86 (22.9)		
Lower third	564 (45.2)	380 (43.5)	184 (49.1)		
Entire	18 (1.4)	13 (1.5)	5 (1.3)		
Tumor size (cm)				0.057	0.811
<3.900	682 (54.6)	479 (54.9)	203 (54.1)		
≥3.900	566 (45.4)	394 (45.1)	172 (45.9)		
Pathological type				2.311	0.510
Well differentiated	40 (3.2)	29 (3.3)	11 (2.9)		
Moderately differentiated	313 (25.1)	229 (26.2)	84 (22.4)		
Poorly differentiated	741 (59.4)	509 (58.3)	232 (61.9)		
Signet ring cell/Mucinous /undifferentiated	154 (12.3)	106 (12.2)	48 (12.8)		
Surgical Method				3.531	0.171
Proximal Gastrectomy	148 (11.9)	111 (12.7)	37 (9.9)		
Distal Gastrectomy	634 (50.8)	430 (49.3)	204 (54.4)		
Total Gastrectomy	466 (37.3)	332 (38.0)	134 (35.7)		
T stage				0.327	0.955
T1	340 (27.2)	236 (27.0)	104 (27.7)		
T2	194 (15.6)	138 (15.8)	56 (14.9)		
T3	394 (31.6)	273 (31.3)	121 (32.3)		
T4	320 (25.6)	226 (25.9)	94 (25.1)		
N stage				1.189	0.756
N0	596 (47.8)	421 (48.2)	175 (46.7)		
N1	242 (19.4)	173 (19.8)	69 (18.4)		
N2	220 (17.6)	151 (17.3)	69 (18.4)		
N3	190 (15.2)	128 (14.7)	62 (16.5)		
TNM stage				1.411	0.494
I	433 (34.7)	304 (34.8)	129 (34.4)		
II	380 (30.4)	273 (31.3)	107 (28.5)		
III	435 (34.9)	296 (33.9)	139 (37.1)		
Neural invasion				1.799	0.180
Negative	347 (27.8)	233 (26.7)	114 (30.4)		
Positive	901 (72.2)	640 (73.3)	261 (69.6)		
Lymphovascular invasion				0.186	0.667
Negative	584 (46.8)	412 (47.2)	172 (45.9)		
Positive	664 (53.2)	461 (52.8)	203 (54.1)		
LNH				0.016	0.899
<30	1021 (81.8)	715 (81.9)	306 (81.6)		
≥30	227 (18.2)	158 (18.1)	69 (18.4)		
TP (g/L)				0.727	0.394
<67.100	763 (61.1)	527 (60.4)	236 (62.9)		
≥67.100	485 (38.9)	346 (39.6)	139 (37.1)		
HB (g/L)				0.601	0.438
<104	188 (15.1)	136 (15.6)	52 (13.9)		
≥104	1060 (84.9)	737 (84.4)	323 (86.1)		
ALB (g/L)				0.221	0.639
<40.700	728 (58.3)	513 (58.8)	215 (57.3)		
≥40.700	520 (41.7)	360 (41.2)	160 (42.7)		
CEA (ng/ml)				0.983	0.321
<6.450	1108 (88.8)	770 (88.2)	338 (90.1)		
≥6.450	140 (11.2)	103 (11.8)	37 (9.87)		
AFP (ng/ml)				0.270	0.603
<3.315	779 (62.4)	549 (62.9)	230 (61.3)		
≥3.315	469 (37.6)	324 (37.1)	145 (38.7)		
CA125 (U/ml)				0.048	0.827
<16.275	1046 (83.8)	733 (84.0)	313 (83.5)		
≥16.275	202 (16.2)	140 (16.0)	62 (16.5)		
CA19-9 (U/ml)				1.051	0.305
<24.735	1039 (83.3)	733 (84.0)	306 (81.6)		
≥24.735	209 (16.7)	140 (16.0)	69 (18.4)		
Neutrophils (10^9^/L)				2.298	0.130
<8.960	1001 (80.2)	710 (81.3)	291 (77.6)		
≥8.960	247 (19.8)	163 (18.7)	84 (22.4)		
Monocytes (10^9^/L)				1.038	0.308
<0.280	384 (30.8)	261 (29.9)	123 (32.8)		
≥0.280	864 (69.2)	612 (70.1)	252 (67.2)		
Lymphocytes (10^9^/L)				1.356	0.244
<1.430	743 (59.5)	529 (60.6)	214 (57.1)		
≥1.430	505 (40.5)	344 (39.4)	161 (42.9)		
Eosinophils (10^9^/L)				1.493	0.222
<0.060	636 (51.0)	435 (49.8)	201 (53.6)		
≥0.060	612 (49.0)	438 (50.2)	174 (46.4)		
Basophils (10^9^/L)				0.233	0.629
<0.030	736 (59.0)	511 (58.5)	225 (60.0)		
≥0.030	512 (41.0)	362 (41.5)	150 (40.0)		
RBC (10^12^/L)				0.004	0.949
<4.480	664 (53.2)	465 (53.3)	199 (53.1)		
≥4.480	584 (46.8)	408 (46.7)	176 (46.9)		
Platelet (10^9^/L)				2.213	0.137
<281	1056 (84.6)	730 (83.6)	326 (86.9)		
≥281	192 (15.4)	143 (16.4)	49 (13.1)		
Liver metastases				0.056	0.813
No	1121 (89.8)	783 (89.7)	338 (90.1)		
Yes	127 (10.2)	90 (10.3)	37 (9.9)		

### Predictive factors selection and development of nomogram

**Figure 1:**
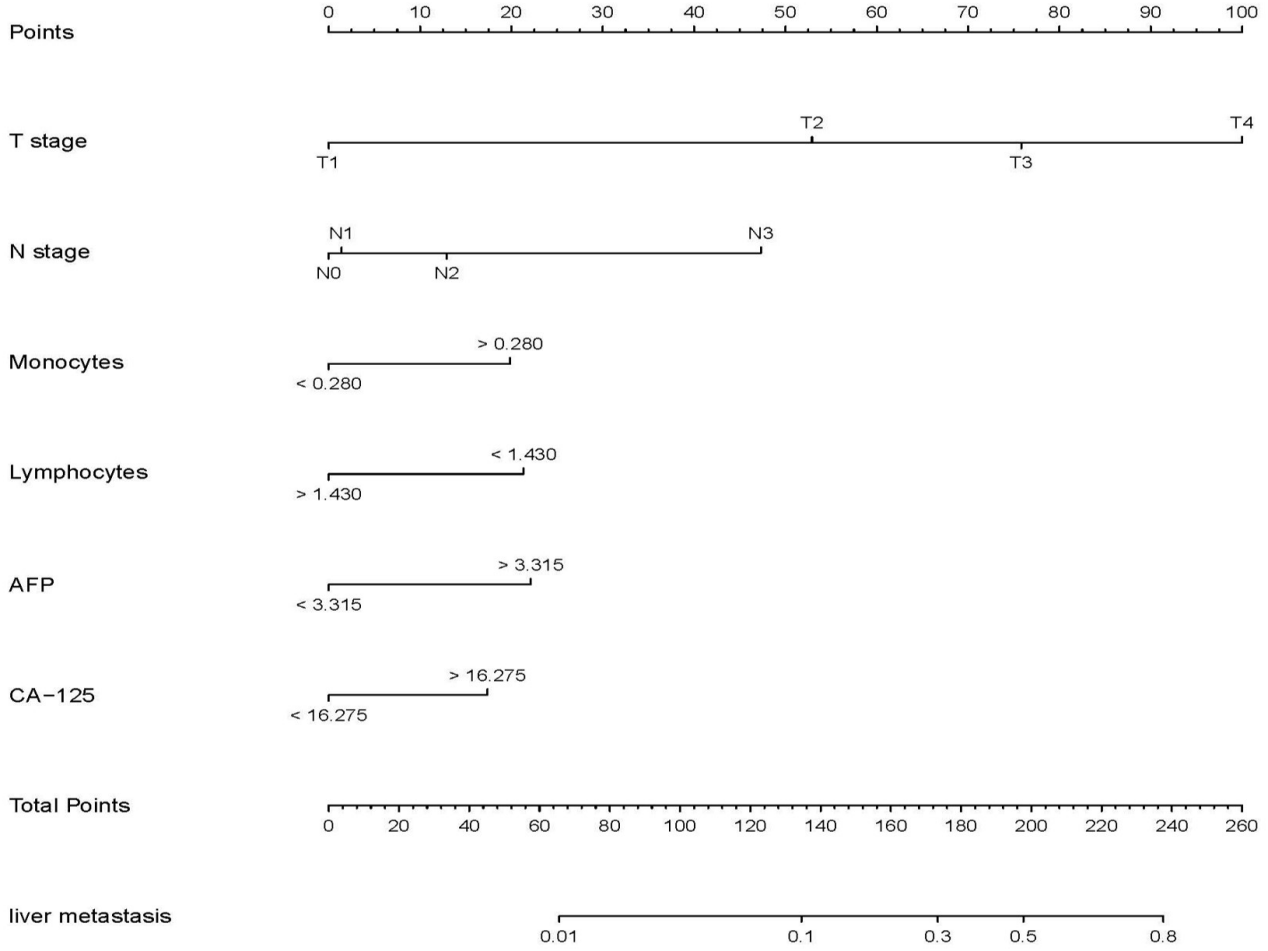
Nomogram for predicting metachronous liver metastasis in patients with gastric cancer.

Univariable analysis in the training set was conducted to identify the potential predictors for MLM in gastric cancer patients. Univariate analysis showed that age, tumor size, pathological type, surgical method, T stage, N stage, TNM stage, neural invasion, lymphatic vascular invasion, LNH, TP, HB, ALB, CEA, CA19-9, CA125, AFP levels, monocyte, lymphocyte, RBC and platelet counts were risk factors for MLM in gastric cancer. In all associated features (p<0.1), potential predictors in the training data were selected by multivariable logistic regression. The multivariable logistic regression analysis revealed that T stage, N stage, monocyte count, lymphocyte count, AFP level, and CA125 level served as independent predictors for MLM, as shown in **Table 2**. Then we developed the nomogram based on the above predictors (**Figure 1**).

**Table 2:** Univariate and multivariate logistic regression in the training cohort.

Characteristics	Non-LM group (n=783) (%)	LM group (n=90) (%)	Univariate analysis OR (95% CI)	P	Multivariate analysis OR (95% CI)	P
Gender			0.716 (0.401-1.278)	0.258		
Male	612 (78.2)	75 (83.3)				
Female	171 (21.8)	15 (16.7)				
Age (year)			1.622 (1.015-2.590)	0.043		
<57	331 (42.3)	28 (31.1)				
≥57	452 (57.7)	62 (68.9)				
Tumor location			1.057 (0.824-1.357)	0.661		
Upper third	235 (30.0)	30 (33.3)				
Middle third	196 (25.0)	19 (21.1)				
Lower third	346 (44.2)	34 (37.8)				
Entire	6 (0.8)	7 (7.8)				
Tumor size (cm)			4.958 (2.957-8.314)	<0.001		
<3.900	459 (58.6)	20 (22.2)				
≥3.900	324 (41.4)	70 (77.8)				
Pathological type			1.491 (1.071-2.076)	0.018		
Well differentiated	29 (3.7)	0 (0.0)				
Moderately differentiated	215 (27.4)	14 (15.6)				
Poorly differentiated	443 (56.6)	66 (73.3)				
Signet ring cell/Mucinous /undifferentiated	96 (12.3)	10 (11.1)				
Surgical Method			1.669 (1.174-2.373)	0.004		
Proximal Gastrectomy	102 (13.0)	9 (10.0)				
Distal Gastrectomy	398 (50.8)	32 (35.6)				
Total Gastrectomy	283 (36.2)	49 (54.4)				
T stage			3.466 (2.517-4.774)	<0.001	2.487 (1.750-3.533)	<0.001
T1	235 (30.0)	1 (1.1)				
T2	133 (17.0)	5 (5.6)				
T3	247 (31.5)	26 (28.9)				
T4	168 (21.5)	58 (64.4)				
N stage			2.525 (2.044-3.118)	<0.001	1.815 (1.437-2.292)	<0.001
N0	406 (51.8)	15 (16.7)				
N1	162 (20.7)	11 (12.2)				
N2	133 (17.0)	18 (20.0)				
N3	82 (10.5)	46 (51.1)				
TNM stage			5.106 (3.376-7.724)	<0.001		
I	301 (38.4)	3 (3.3)				
II	256 (32.7)	17 (18.9)				
III	226 (28.9)	70 (77.8)				
Neural invasion			4.811 (2.191-10.566)	<0.001		
Negative	226 (28.9)	7 (7.78)				
Positive	557 (71.1)	83 (92.2)				
Lymphovascular invasion			4.733 (2.708-8.271)	<0.001		
Negative	396 (50.6)	16 (17.8)				
Positive	387 (49.4)	74 (82.2)				
LNH			1.648 (0.991-2.740)	0.054		
<30	648 (82.8)	67 (74.4)				
≥30	135 (17.2)	23 (25.6)				
TP (g/L)			0.588 (0.365-0.948)	0.029		
<67.100	463 (59.1)	64 (71.1)				
≥67.100	320 (40.9)	26 (28.9)				
HB (g/L)			0.377 (0.230-0.619)	<0.001		
<104	109 (13.9)	27 (30.0)				
≥104	674 (86.1)	63 (70.0)				
ALB (g/L)			0.514 (0.317-0.833)	0.007		
<40.700	448 (57.2)	65 (72.2)				
≥40.700	335 (42.8)	25 (27.8)				
CEA (ng/ml)			3.725 (2.230-6.221)	<0.001		
<6.450	706 (90.2)	64 (71.1)				
≥6.450	77 (9.83)	26 (28.9)				
AFP (ng/ml)			1.718 (1.108-2.664)	0.016	2.125 (1.296-3.485)	0.003
<3.315	503 (64.2)	46 (51.1)				
≥3.315	280 (35.8)	44 (48.9)				
CA125 (U/ml)			2.706 (1.659-4.412)	<0.001	1.807 (1.042-3.133)	0.035
<16.275	671 (85.7)	62 (68.9)				
≥16.275	112 (14.3)	28 (31.1)				
CA19-9 (U/ml)			2.878 (1.771-4.677)	<0.001		
<24.735	672 (85.8)	61 (67.8)				
≥24.735	111 (14.2)	29 (32.2)				
Neutrophils (10^9^/L)			0.644 (0.342-1.213)	0.173		
<8.960	632 (80.7)	78 (86.7)				
≥8.960	151 (19.3)	12 (13.3)				
Monocytes (10^9^/L)			2.106 (1.202-3.691)	0.009	2.081 (1.105-3.918)	0.023
<0.280	245 (31.3)	16 (17.8)				
≥0.280	538 (68.7)	74 (82.2)				
Lymphocytes (10^9^/L)			0.526 (0.323-0.857)	0.010	0.452 (0.259-0.789)	0.005
<1.430	463 (59.1)	66 (73.3)				
≥1.430	320 (40.9)	24 (26.7)				
Eosinophils (10^9^/L)			1.407 (0.905-2.188)	0.129		
<0.060	397 (50.7)	38 (42.2)				
≥0.060	386 (49.3)	52 (57.8)				
Basophils (10^9^/L)			0.799 (0.509-1.255)	0.330		
<0.030	454 (58.0)	57 (63.3)				
≥0.030	329 (42.0)	33 (36.7)				
RBC (10^12^/L)			0.507 (0.319-0.806)	0.004		
<4.480	404 (51.6)	61 (67.8)				
≥4.480	379 (48.4)	29 (32.2)				
Platelet (10^9^/L)			2.029 (1.223-3.366)	0.006		
<281	664 (84.8)	66 (73.3)				
≥281	119 (15.2)	24 (26.7)				

### Evaluation and clinical application of the nomogram

**Figure 2:**
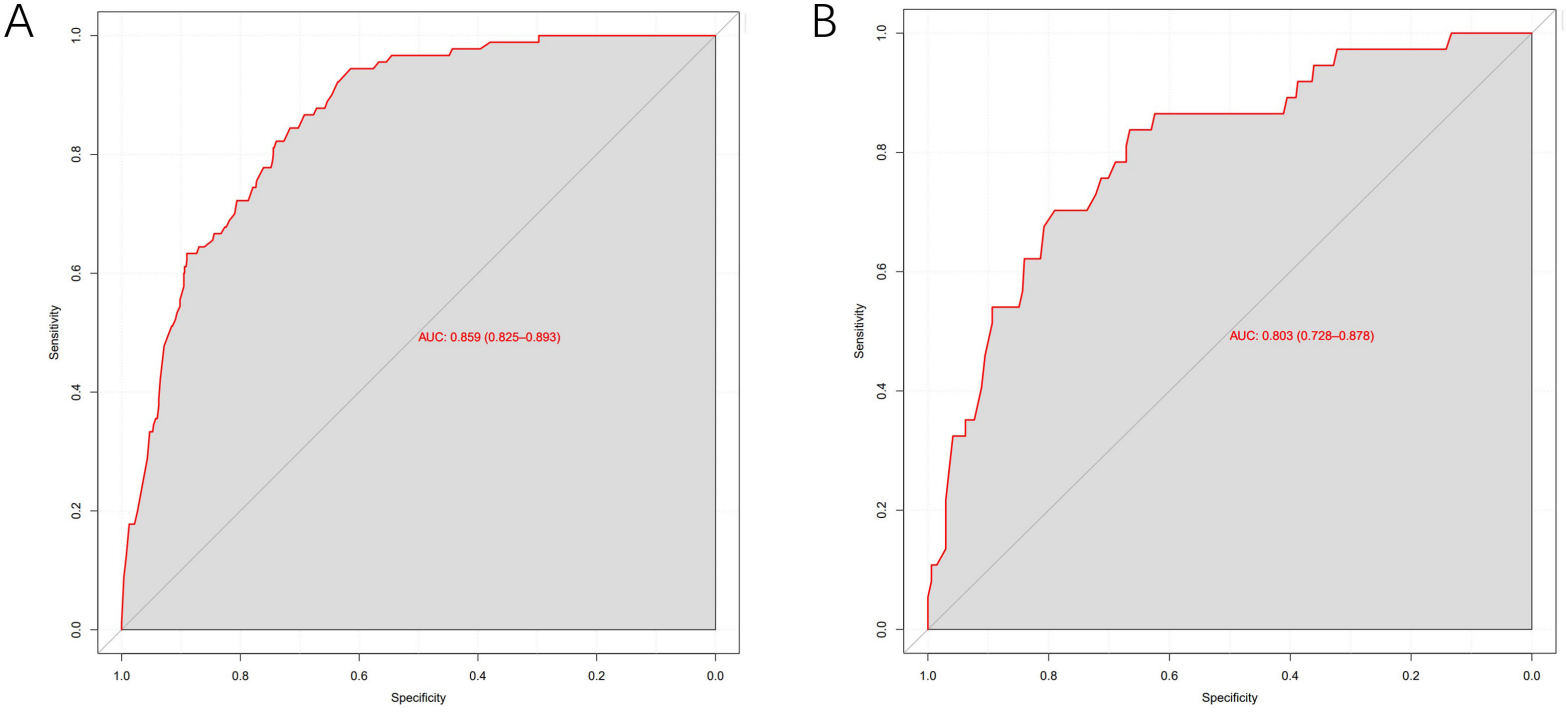
ROC curves of the predictive MLM model in the training cohort **(A)** and validation cohort **(B)**.

**Figure 3:**
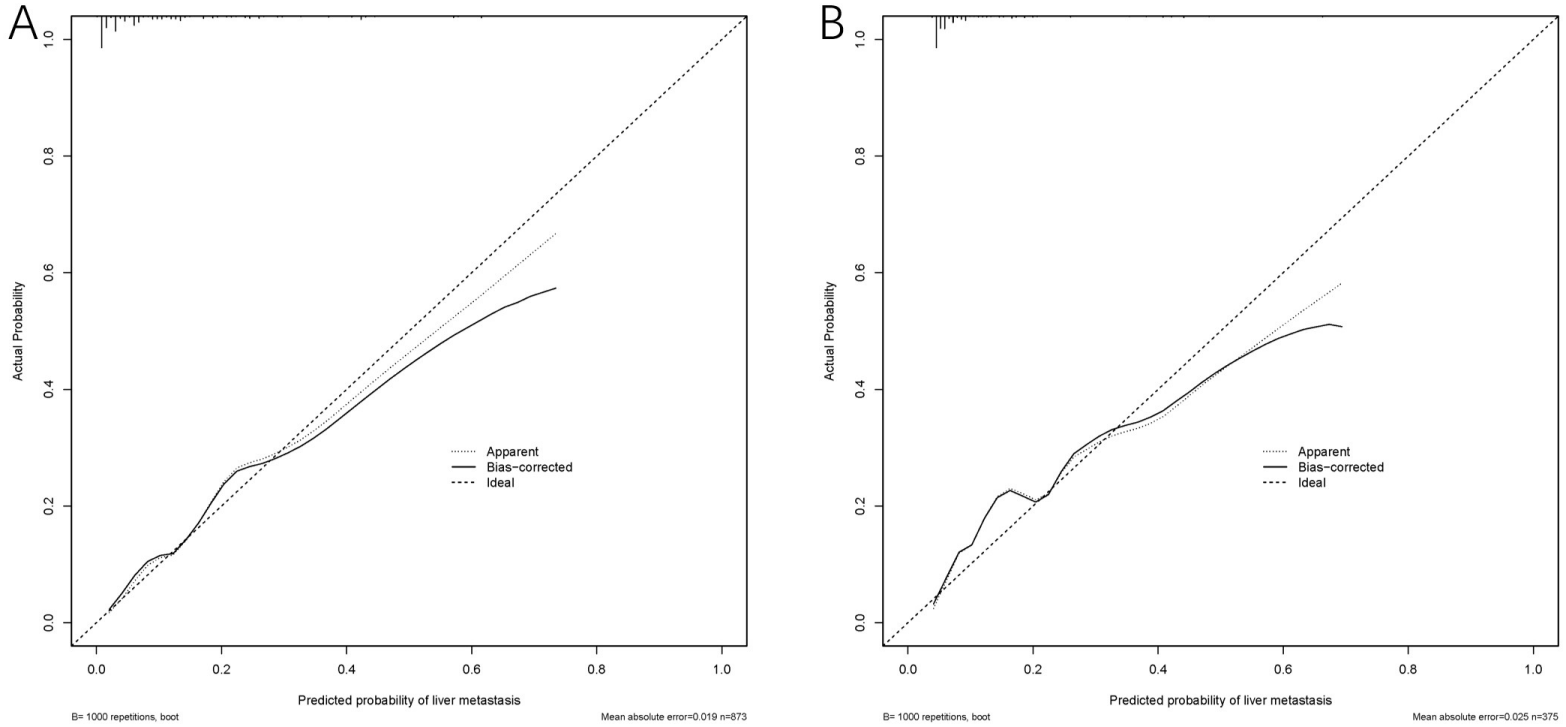
Calibration curves of the MLM nomogram in the cohort. Calibration curve for the training cohort **(A)** and the validation cohort **(B)**.

**Figure 4:**
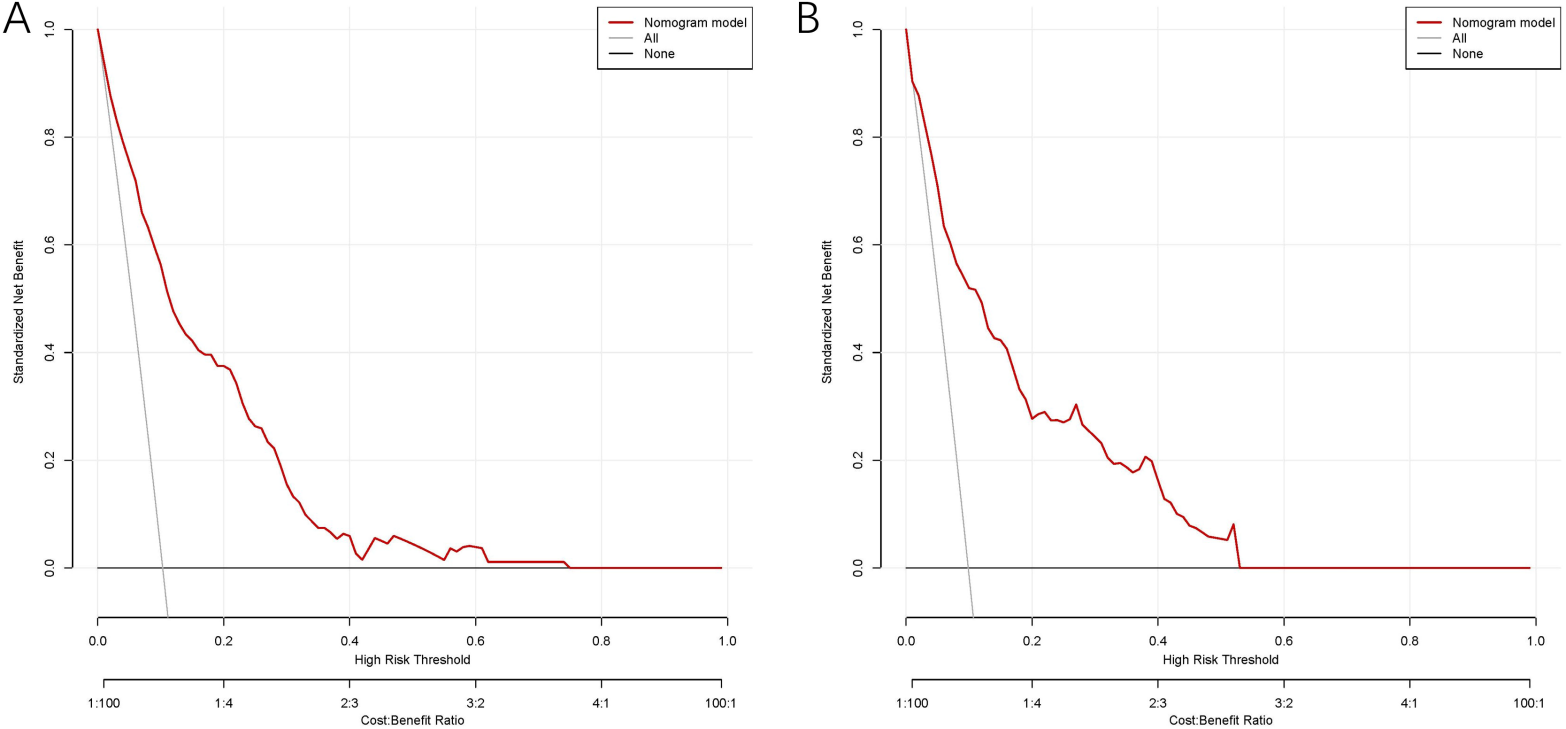
DCA of the nomogram model for predicting MLM in the training cohort **(A)** and validation cohort **(B)**.

The model exhibited strong predictive performance, with AUCs of 0.859 and 0.803 in the training and validation cohorts, respectively (**Figure 2**). Calibration plots indicated excellent agreement between predicted and observed results. The curves aligned closely to the 45-degree reference line, confirming reliable calibration (**Figure 3**). The DCA curves revealed substantial net clinical benefit, highlighting the model’s utility in clinical practice (**Figure 4**). Furthermore, the training cohort conducted an accuracy of 0.748, sensitivity of 0.822, and specificity of 0.739, while the validation cohort exhibited corresponding metrics of 0.683, 0.838, and 0.666, respectively (**Table 3**).

**Table 3:** The predictive performance of the training cohort and the validation cohort.

Model	Accuracy	Sensitivity	Specificity	AUC (95%CI)
Training cohort	0.748	0.822	0.739	0.859 (0.825-0.893)
Validation cohort	0.683	0.838	0.666	0.803 (0.728-0.878)

## Discussion

MLM is closely associated with poor prognosis in gastric cancer, highlighting its critical role in the clinical management of advanced-stage disease (17). However, current research on MLM in gastric cancer remains limited. In this retrospective study, the results showed that T stage, N stage, AFP level, CA125 level, monocyte count, and lymphocyte count were independent predictors of MLM. Furthermore, we developed a nomogram and validated its high accuracy, reliable calibration, and robust clinical utility. Importantly, all variables are routinely obtained during treatment, emphasizing the nomogram’s cost-effectiveness and practical application.

Tumor markers serve as indispensable tools in oncological practice, providing critical insights for both diagnostic evaluation and prognostic stratification of malignancies (18). Recent studies have highlighted the significant value of tumor markers, particularly CEA, CA125, CA19-9, and CA72-4, in the diagnosis and monitoring of gastrointestinal malignancies (19–21). AFP was initially established as a diagnostic biomarker for primary liver cancer (22). In recent years, AFP was recently recognized as an independent prognostic factor for poor outcomes of gastric cancer (23), particularly in AFP-producing gastric cancer (24). Previous studies found that elevated AFP level in gastric cancer was a risk factor for postoperative liver metastasis (25–27), which were consistent with our findings. Study showed that overexpression of AFP in gastric cancer patients significantly inhibited the infiltration of CD8+T cell, could promote liver metastasis by regulating the PTEN/AKT1/SOX5/CES1 signaling axis (28). Moreover, overexpression of AFP upregulated malignancy-related pathways, such as epithelial-mesenchymal transition (EMT) and angiogenesis (29). These findings suggest a potential mechanism through which AFP facilitates MLM in gastric cancer.

CA125, a high-molecular-weight transmembrane glycoprotein, has been established as the gold-standard biomarker for ovarian cancer (30). Emerging evidence has elucidated its prognostic utility in gastrointestinal malignancies, particularly for risk stratification in gastric (31) and colorectal cancer (32). While multiple studies (18, 33) reported CA125 as a predictive factor for postoperative peritoneal metastasis in gastric cancer, only Yang et al. (26) identified CA125 as an independent risk factor for postoperative liver metastasis in gastric cancer, which is consistent with our findings. Currently, the potential role of CA125 promote MLM in gastrointestinal tumor remains unexplored. Marimuthu et al. (34) discovered that CA125 regulated NRP2 via JAK2/STAT1 signaling and induced liver metastasis in pancreatic ductal adenocarcinoma (PDAC). This mechanism may provide a novel research direction for investigating CA125-mediated MLM in gastric cancer. Based on this, we will conduct a multicenter cohort study to further validate the model’s efficacy and accuracy, while incorporating postoperative tumor markers (AFP and CA125) to test its predictive performance.

Notably, to the best of our knowledge, this was the first study to utilize absolute peripheral blood cell counts for predicting MLM in gastric cancer. Monocytes, as precursors of tumor-associated macrophages (TAMs), reflect changes in systemic immune function (35). Elevated monocyte count has been shown to be associated with poor prognosis in gastrointestinal tumors (36, 37). Additionally, Dou et al. (38) reported that monocytes can be used to predict postoperative liver metastasis in colorectal cancer. In our study, elevated monocyte count was identified for the first time as an independent risk factor for MLM in gastric cancer. As key mediators between innate and adaptive immunity, monocytes significantly influence the tumor microenvironment by facilitating immune tolerance, promoting vascular formation, and enhancing tumor cell spread through diverse biological pathways (35). Furthermore, distinct monocyte subpopulations directly contribute to metastatic progression through CXCL2-mediated interactions with circulating tumor cells (39).

As central effectors of adaptive immunity, lymphocytes play a crucial role in antitumor immune responses by inhibiting the progression of various cancers (40, 41). Previous studies demonstrated that lymphocyte count serves as a significant prognostic indicator associated with poor prognosis in gastric cancer (42, 43). Currently, the predictive potential of lymphocytes for MLM in gastrointestinal cancers remains unexplored. In our study, reduced lymphocyte count was identified as an independent predictor of MLM. Mechanistically, tumor cells promote the production of immunosuppressive molecules, potentially resulting in lymphopenia and facilitating immune evasion (44, 45). This decline provides tumor cells, which would normally be eliminated by the immune system, to evade immune surveillance. Consequently, these cells gain increased opportunities to metastasis.

As demonstrated in our results, our study established a novel nomogram to predict MLM in gastric cancer. Although prior studies (25, 27) have elucidated risk factors associated with postoperative liver metastasis, no dedicated predictive model for MLM has been established. While Yang et al. (26) developed a predictive model for postoperative liver metastasis, it lacks specificity for MLM and relies heavily on complex radiological parameters. ​Critically, our model innovatively incorporates preoperative peripheral blood parameters to establish a simple yet robust predictor, demonstrating superior discriminative value.

There were some limitations in our study which must be acknowledged. First of all, it was a retrospective, single-center study, and may have been prone to recall bias as well as loss to follow-up bias. Secondly, different methods could be used to calculate cutoff values, including median value, outliers and quartiles. A comparison of predictive model values based on these varying cutoff criteria was not conducted. Thirdly, the predictive value of postoperative tumor markers and peripheral blood cells were not evaluated.

In conclusion, our study developed and validated a novel, cost-effective, and easily accessible nomogram. Besides being predicted with conventional parameters, our model incorporated preoperative peripheral blood monocyte and lymphocyte counts to predict MLM risk in gastric cancer patients, which could enhance the model’s accuracy and predictive value.

## Data Availability

The raw data supporting the conclusions of this article will be made available by the authors, without undue reservation.
